# Wenxin-Keli Regulates the Calcium/Calmodulin-Dependent Protein Kinase II Signal Transduction Pathway and Inhibits Cardiac Arrhythmia in Rats with Myocardial Infarction

**DOI:** 10.1155/2013/464508

**Published:** 2013-05-28

**Authors:** Yanwei Xing, Yonghong Gao, Jianxin Chen, Haiyan Zhu, Aiming Wu, Qing Yang, Fei Teng, Dong-mei Zhang, Yanhui Xing, Kuo Gao, Qingyong He, Zhenpeng Zhang, Jie Wang, Hongcai Shang

**Affiliations:** ^1^Guang'anmen Hospital, Chinese Academy of Chinese Medical Sciences, Beijing 100053, China; ^2^The Key Laboratory of Chinese Internal Medicine of the Ministry of Education, Dongzhimen Hospital Affiliated to Beijing University of Chinese Medicine, Beijing 100700, China; ^3^Beijing University of Chinese Medicine, Beijing 100029, China; ^4^College of Traditional Chinese medicine, Ningxia Medical University, Yinchuan 750004, China; ^5^Institute of Information on Traditional Chinese Medicine, China Academy of Chinese Medical Sciences, Beijing 100700, China; ^6^Tianjin University of Traditional Chinese Medicine, Tianjin 300193, China

## Abstract

Wenxin-Keli (WXKL) is a Chinese herbal compound reported to be of benefit in the treatment of cardiac arrhythmia, cardiac inflammation, and heart failure. Amiodarone is a noncompetitive inhibitor of the **α**- and **β**-adrenergic receptors and prevents calcium influx in the slow-response cells of the sinoatrial and atrioventricular nodes. Overexpression of Ca^2+^/calmodulin-dependent protein kinase II (CaMKII) in transgenic mice results in heart failure and arrhythmias. We hypothesised that administration of WXKL and amiodarone can reduce the incidence of arrhythmias by regulating CaMKII signal transduction. A total of 100 healthy Sprague Dawley rats were used in the study. The rats were randomly divided into four groups (a sham group, a myocardial infarction (MI) group, a WXKL-treated group, and an amiodarone-treated group). A myocardial infarction model was established in these rats by ligating the left anterior descending coronary artery for 4 weeks. Western blotting was used to assess CaMKII, p-CaMKII (Thr-286), PLB, p-PLB (Thr-17), RYR2, and FK binding protein 12.6 (FKBP12.6) levels. The Ca^2+^ content in the sarcoplasmic reticulum (SR) and the calcium transient amplitude were studied by confocal imaging using the fluorescent indicator Fura-4. In conclusion, WXKL may inhibit heart failure and cardiac arrhythmias by regulating the CaMKII signal transduction pathway similar to amiodarone.

## 1. Introduction

Cardiovascular diseases are the most common threat to human health worldwide and are the leading cause of morbidity in human populations. Epidemiological data show that at present the number of patients with heart failure has increased to 22.5 million globally and is still increasing at a rate of 2 million patients per year. The 5-year survival rate of patients with heart failure is similar to that of patients with malignant tumours, and mortality continues to increase despite advances in our understanding of the underlying mechanisms of the disease and the development of new treatments. Currently, more than a quarter of a million patients die of heart failure annually in the United States [1]. The risk of sudden cardiac death in heart failure patients is six to nine times that of the general population, and approximately half of these patients die of ventricular arrhythmias. Although drugs that inhibit the adrenergic and renin-angiotensin aldosterone systems have improved the survival of individual patients receiving treatment, deaths from heart failure increased by 28% between the years 1994 and 2004 [2–4]. Treating arrhythmias in patients with structural heart disease using ion channel antagonist drugs does not reduce mortality [5, 6]. These data highlight the importance of finding suitable agents for treating both heart failure and arrhythmias.

In the past, antiarrhythmic drug research mainly targeted the various types of ion channels in the cell membrane. With the rapid development of the biological sciences, finding new targets for anti-arrhythmic treatment at the level of cellular signal transduction has become a promising new avenue for research. The use of beta blockers for the management of heart failure is one example in which targeting a signalling pathway rather than an individual family of ion channels has proved effective. CaMKII has emerged as a key intracellular signalling molecule, that is, increasingly being recognised as a critical player in cardiac disease and arrhythmia. As we learn more about CaMKII and its effects in the heart, it appears that it also may be a potential therapeutic target [7]. Intracellular CaMKII signal transduction pathways play a central role in the regulation of intracellular calcium. CaMKII is a multifunctional serine/threonine protein kinase, and its expression is increased in both ischemic and dilated cardiomyopathies [8, 9]. CaMKII regulates a wide variety of downstream targets in the heart, including sarcolemmal ion channels (e.g., L-type Ca and Na channels), SR Ca release channels, and PLB, and therefore is important in regulating SR Ca release and Ca reuptake. Thus, CaMKII is critical for the fine tuning of cardiac excitation-contraction coupling (ECC) [10].

Under physiological conditions, CaMKII phosphorylation can keep the calcium channel open and keep intracellular calcium at a moderate level [11]. CaMKII mediates the activity of the L-type calcium channel (LTCC) and RyR via phosphorylation-dependent events, which are integral to normal ECC. When a cardiomyocyte is depolarised by a propagating action potential, calcium enters the cell via the LTCC. This initial calcium entry activates the ryanodine receptor, resulting in the release of calcium from the SR by a process termed calcium-induced calcium release. Release of calcium from the SR accounts for the majority of the intracellular calcium, that is, necessary for contraction and other functions of the cardiomyocyte. The majority of the cytosolic calcium is removed by sarcoplasmic reticulum calcium pump (SERCA), which is negatively regulated by PLB. The return of the cytosolic calcium to basal levels signals the beginning of diastole [4]. Heart failure is associated with excess CaMKII activity. This excess CaMKII activity results in hyperphosphorylation of the LTCC, RyR, SERCA, and PLB proteins, which impairs cardiac function and predisposes the cardiac myocytes to after depolarisations. Hyperphosphorylation of a subunit of the LTCC results in increased I_Ca_, which is a factor in the predisposition of the cardiac myocytes to EADs (which occurs at phases 2 and 3 of the action potential). Hyperphosphorylation events at the SR lead to the depletion of SR calcium stores, which results in impaired cytosolic calcium transients that in turn cause systolic and diastolic dysfunction. Furthermore, hyperphosphorylation of RyR2 results in an SR calcium leak that can lead to a net inward Na current via sodium-calcium exchanger (NCX) resulting in delayed after depolarisations (i.e., occurring after the completion of repolarisation). However, recent studies have found that, under conditions of heart failure, the ability of protein kinase A (PKA) to regulate the phosphorylation of the RyR2 protein is dependent on CaMKII activity [12, 13]. FKBP12.6 is a regulator of RyR2 channel activity, and binding of FKBPl2.6 to RyR2 causes the channel to maintain its closed state. In heart failure, enhanced sympathetic nerve excitation leads to increased CaMKII activity, which results in hyperphosphorylation of RyR2. This hyperphosphorylation causes the dissociation of FKBP12.6 from RyR2, which opens the channel, and the resulting increase in Ca^2+^ leakage causes triggered beat activity to increase [14–17].

WXKL was developed at Guang'anmen Hospital, a facility of the Chinese Academy of Chinese Medical Sciences, and was the first Chinese-developed anti-arrhythmic medicine to be approved by the Chinese state. In clinical applications, WXKL has been shown to be effective in the treatment of chronic heart failure and arrhythmia. The main ingredients of WXKL consist of *Codonopsis*, *Polygonatum*, *Panax*, nard, and amber. A large number of clinical trials have confirmed that WXKL can increase coronary blood flow, reduce myocardial oxygen consumption, enhance myocardial compliance, improve myocardial hypoxia tolerance, relieve anterior and posterior cardiac loading, reduce myocardial tissue damage in patients with high blood pressure, and reduce the occurrence of arrhythmia [18]. Findings also indicate that WXKL produces atrial-selective depression of I_Na_-dependent parameters in canine isolated coronary-perfused preparations via a unique mechanism and is effective in both suppressing AF and preventing its induction [19].

In the present study, the hypothesis that WXKL can reduce the incidence of arrhythmias by regulating the CaMKII signal transduction pathway was tested *in vitro *and *in vivo*, and its antiarrhythmic effects were compared to those of amiodarone. Amiodarone is a noncompetitive inhibitor of the *α*- and *β*-adrenergic receptors and prevents calcium influx in slow-response cells. Amiodarone can expand blood vessels and slow the heart rate to reduce myocardial ischemia. Amiodarone can also directly prolong the duration of the action potential, the repolarisation time, and the refractory period through inhibition of the outward potassium current, and it is categorised as a class III anti-arrhythmic drug. It has been reported that the ability of amiodarone to suppress the activity of CaMKII may be a component of its anti-arrhythmic therapeutic mechanism. However, the multisystem side effects associated with amiodarone limit its long-term use in patients.

## 2. Material and Methods

### 2.1. WXKL Compound

WXKL, consisting of* Rhizoma nardostachyos*, *Codonopsis*, *Notoginseng*, amber, and *Rhizoma Polygonati*, was provided by the BuChang Group, Xi'An, China. According to the national pharmacopoeia (National Pharmacopoeia Committee, 2005), the total amount of notoginseng saponin R1 (C_47_H_80_O_18_), ginseng saponin Rg1 (C_42_H_72_O_14_), and ginseng saponin Rb1 (C_54_H_92_O_23_) should not be less than 17 mg per bag (9 g). The powdered WXKL compound was dissolved in distilled water prior to use.

### 2.2. Animal Grouping and Administration of Drugs

One hundred male Sprague Dawley rats (160 ± 20 g), purchased from the animal laboratory of the Academy of Medical Sciences, Beijing, China, were initially divided into two groups: a sham group (*N* = 25) and an MI group (*N* = 75). MI and sham rats were fed normally for 2 weeks before being prescreened by twelve-lead electrocardiogram (ECG). The MI rats with 6–8 leads having q waves were included in the study. The 75 MI rats were randomly assigned to three treatment groups: the MI group (*N* = 25), in which the rats were treated with the vehicle alone (distilled water, 1 mL/kg/day) for oral administration; the WXKL group (*N* = 25), in which the rats were treated with the WXKL compound (4 g/kg/day) for oral administration; and the amiodarone group (*N* = 25), in which the rats were treated with amiodarone (30 mg/kg/day) for oral administration. All animals used in this study received humane care in compliance with the National Institutes of Health Guide for the Care and Use of Laboratory Animals.

### 2.3. Establishment of the Myocardial Infarction Model and Sham-Operated Rats

The rats were anaesthetised by intraperitoneal injection of a 1% solution of sodium pentobarbital (50 mg/kg). The procedures performed consisted of endotracheal intubation, positive pressure ventilation, preoperative recording by twelve-lead ECG, one-lead monitoring, local skin disinfection, chest opening, thoracotomy device setup and opening of the pericardium, and ligation of the pulmonary cone and the left atrial appendage 2-3 mm from the bottom of the left anterior descending coronary artery ligation. In the sham group, the left anterior descending artery was not ligated. Additional twelve-lead ECG recordings were made postoperatively. The rats were fed normally for 4 weeks before being euthanised and dissected to isolate the heart for the subsequent experiments.

### 2.4. Histological Examination

Rat heart samples were cut into transverse sections and stained with haematoxylin and eosin (H&E).

### 2.5.   Inhibitor of CaMKII

Treatment with KN93 (Sigma Inc.), a specific inhibitor of CaMKII, was also found to significantly reduce the Ca transient amplitudes in cardiac myocytes, while treatment with KN92 (Sigma Inc.), which has the same structure as KN93 but no CaMKII-inhibiting activity, had no effect.

### 2.6. Western Blot Analysis

All animals were euthanised after 4 weeks of drug administration, and their hearts were immediately harvested and stored in liquid nitrogen until Western blot analyses were performed. The following antibodies were used: rabbit polyclonal anti-CaMKII (1:500, Santa Cruz Biotechnology Inc.), rabbit polyclonal anti-phospho-CaMKII (Ser-286) (1:1000, Cell Signaling Technology Inc.), antiphospholamban (1:1000, Cell Signaling Technology Inc.), polyclonal anti-phospho-phospholamban (Ser-17) (1:1000, Santa Cruz Biotechnology Inc.), rabbit polyclonal anti-ryanodine receptor 2 (1:1000, Millipore Corporation.), and rabbit polyclonal anti-FKBP12.6 (1:500, Santa Cruz Biotechnology Inc.). Proteins were separated by 10% SDS-PAGE and transferred to nitrocellulose membranes, which were then incubated with antibodies at 4°C. The membranes were further incubated with horseradish peroxidase-conjugated anti-rabbit IgG (1:15,000) for 2 hours at room temperature. ECL visualisation was performed, and the Gene Gnome Gel Imaging System (Syngene Co.) was used to capture the resulting images. Image J (NIH image, Bethesda, MD, USA) was used to analyse the gel images.

### 2.7. Rat Cardiac Myocyte Isolation

Excised rat hearts were mounted on a Langendorff perfusion apparatus and were retrogradely perfused with a nominally Ca-free Tyrode's solution (137 mM NaCl, 5.4 mM KCl, 21.2 mM MgCl_2_, 20 mM HEPES, 1.2 mM NaH_2_PO_4_·2H_2_O, 10 mM glucose, and 10 mM taurine) for 4 minutes at 37°C (pH 7.35). The hearts were then perfused with a digestion solution (25 *μ*M CaCl_2_, 10 mM BDM, 1 mg/mL taurine, 1 mg/mL BSA, and 22-23 mg/mL type II collagenase enzyme (USA, Worthington, 47K9848)) for 20 minutes, at which point the heart became flaccid. Ventricular tissue was removed and cut into small pieces. The tissue was then dispersed until no solid cardiac tissue was left by intensively mixing the myocardial tissue and the enzymatic digestion solution at 37°C for 3 minutes using a constant temperature shaker at 30–50 RPM. To ensure thorough digestion, Ca reintroduction was performed through stepwise increases in Ca concentration from 25 *μ*M to 500 *μ*M. Following their isolation, cardiac myocytes were plated onto superfusion chambers that were coated with laminin to allow cell adhesion. The plated cells were then immediately subjected to physiological analysis. The individual ventricular myocytes selected for study were rod-shaped and had clear striations and a smooth, glossy surface. 

### 2.8. Confocal Imaging

 To record single-cell calcium transients, myocardial cells were transferred to special laser confocal petri dishes (MstTek, P35G, 0.16 mm–0.19 mm thickness, P35G-1.5-14-C) with a 2 mL volume of extracellular fluid containing fluo-4 (F14201, Invitrogen). Measurements were made using a Zeiss LSM-510 inverted confocal microscope (Carl Zeiss, Oberkochen, Germany. Lens: Plan-Neofluar 40x/1.3 oil, numerical aperture of 1.25). All image data were collected in the line-scanning mode along the long axis of the myocyte and with laser excitation at a wavelength of 488 nm. The Ca^2+^ level is reported as *F*/*F*0, where *F*0 is the resting or diastolic fluo-4 fluorescence. The stimulation frequency was set at 0.25 Hz, and the pulse width was 4 s. This type of electrical stimulation causes cell membrane depolarisation, which leads to opening of the LTCC and, therefore, inward calcium ion flow. This inward flow of calcium causes the SR to release large amounts of calcium ions, and this further induces myocardial cell shrinkage. At this point, the calcium concentration increases in the cell become calcium transients.

### 2.9. Determination of SR Ca^2+^ Content

Myocytes were field stimulated at 0.5 Hz, and their SR Ca^2+^ content was assessed by measurement of the amplitude of caffeine-induced Ca^2+^ transients [20]. 

### 2.10. Arrhythmia Induction and WXKL Treatment *In Vivo*

We randomly selected five rats from each experimental group (the sham group, the MI group, the WXKL group, and the amiodarone group). The rats were anaesthetised using 1% sodium pentobarbital as described previously [21], and an equivalent of lead I ECG recording was performed. After stabilisation of the subject, a control ECG was recorded for 5 minutes. This was followed by an intraperitoneal injection of ISO (3 mg/kg body weight) and a subsequent recording period of 10 minutes. During this period, the ECG was analysed for ISO-induced arrhythmias.

### 2.11. Statistical Methods

All experimental data were expressed as the mean ± SD. The data were statistically evaluated using one-way analysis of variance (ANOVA), and a post hoc analysis was performed using Fisher's least significant difference (LSD) test. The SPSS computer program (version 17.0) was used for the analyses. A probability of *P* < 0.05 was considered statistically significant.

## 3. Results

### 3.1. Effects of WXKL on Survival Rate and Heart Weight/Body Weight Ratio at 4 Weeks after Treatment

**Figure 1:**
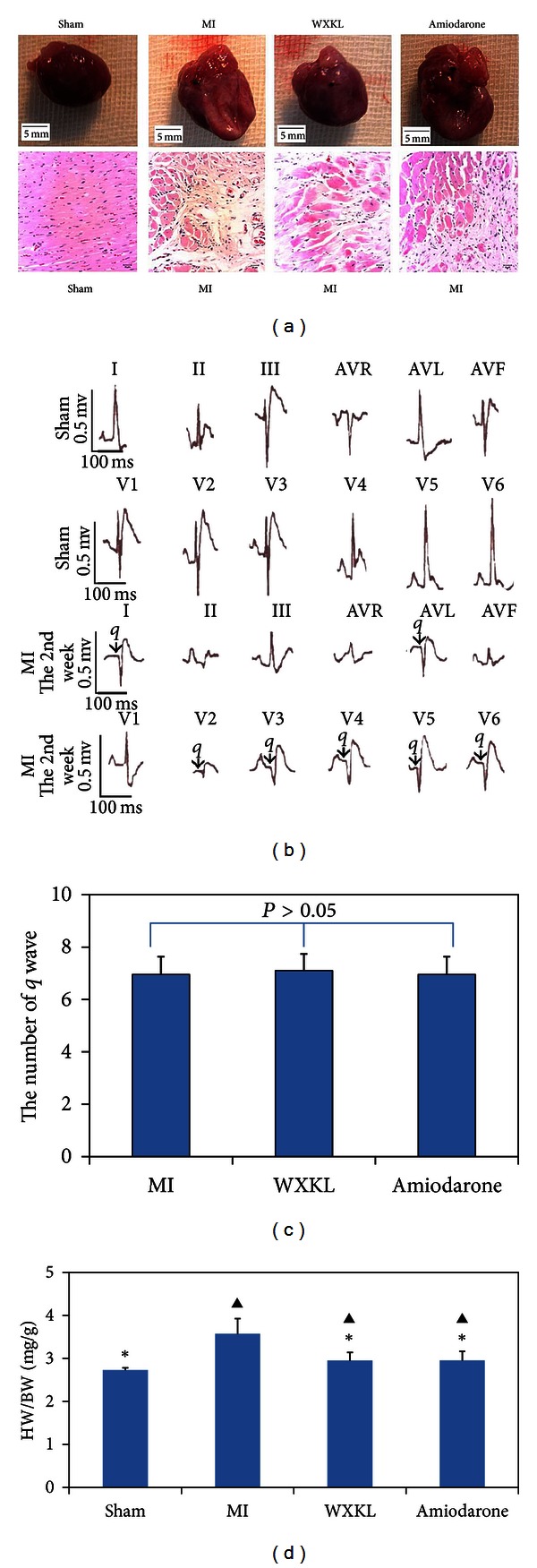
Heart preparation and pathological sections from normal and MI rats. (a) Heart preparations (top (1–4)) and pathological sections (bottom (5–8)) from the sham group, the MI group, the WXKL group, and the amiodarone group. (b) ECG recordings of the sham group and the MI group at 2 weeks after the operation. (c) The average number of q waves in rats from the MI group, the WXKL group, and the amiodarone group. (d) HW (heart weight): BW (body weight) ratios in the sham group (*n* = 25), the MI group (*n* = 23), the WXKL group (*n* = 25), and the amiodarone group (*n* = 25). (*P < 0.05 versus the MI group, ^▲^ *P* < 0.05 versus the sham group).

After treatment for 4 weeks, deaths had occurred only among the MI group, and the survival rates of the MI group (*n* = 23), the sham group (*n* = 25), the WXKL group (*n* = 25), and the amiodarone group (*n* = 25) were therefore 92%, 100%, 100%, and 100%, respectively. No significant differences in survival were observed among the four groups. As shown in Figure 1, long-term treatment with either WXKL or amiodarone significantly reduced the heart weight/body weight ratio (*P* < 0.05).

### 3.2. Posttreatment Assessment of Cardiac Structure and Function by Echocardiography

**Figure 2:**
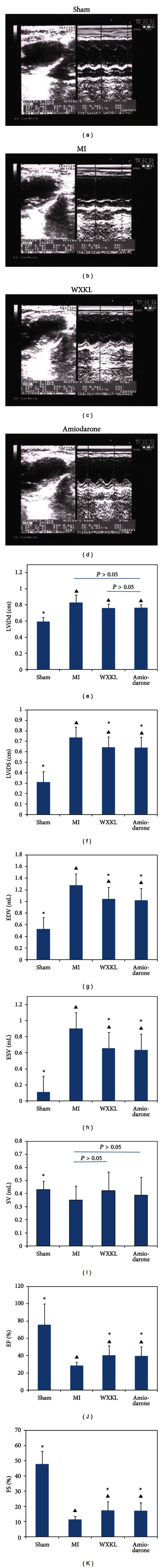
Typical echocardiography images from the sham group (a), the MI group (b), the WXKL group (c), and the amiodarone group (d). At the 4th week of WXKL and amiodarone administration, cardiac structure and function were measured in each group by echocardiography. We evaluated cardiac systolic and diastolic functions by measuring the following variables: left ventricular end-diastolic dimension (LViDd) (e), left ventricular end-systolic dimension (LViDs) (F), end-diastolic volume (EDV) (g), end-systolic volume (ESV) (h), stroke volume (SV) (i), ejection fraction (EF) (j), and fractional shortening (FS) (k). Treatment with either WXKL or amiodarone improved systolic function. The rats of the sham group (*n* = 25) and the MI group (*n* = 23) were treated with vehicle (distilled water) alone (1 mL/kg/day); the WXKL group (*n* = 25) were treated with 4 g/kg/day WXKL; and the amiodarone group (*n* = 25) were treated with 30 mg/kg/day amiodarone. (*P < 0.05 versus the MI group, ^▲^ *P* < 0.05 versus the sham group).

 We evaluated cardiac systolic function by a combination of ECG measurements that included the EF, the FS, EDV, ESV, the left ventricular end-diastolic dimension (LVDd), the LVDs, and the stroke volume (SV). Compared with the MI group (*n* = 23), the EF and FS measurements were elevated in the WXKL group (*n* = 25) and the amiodarone group (*n* = 25) (*P* < 0.05), while the EDV, ESV, and LVDs measurements were lowered (*P* < 0.05). Although the measurements obtained for LVDd displayed a decreasing trend in both the WXKL group and the amiodarone group versus the MI group, the difference was not statistically significant (*P* > 0.05). Compared with those of the sham group, the EF and FS measurements obtained from the MI group, the WXKL group, and the amiodarone group were reduced (*P* < 0.05), while the EDV, ESV, LVDd, and LVDs measurements were increased (*P* < 0.05) (Figure 2).

### 3.3. Effects of WXKL on Expression of CaMKII and Related Proteins

**Figure 3:**
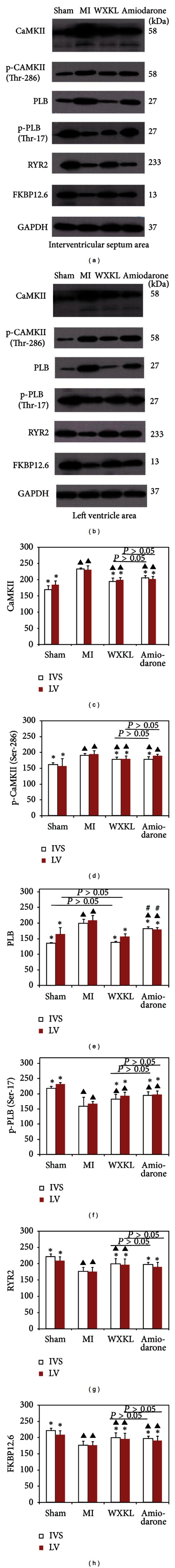
The expression levels of CaMKII and related proteins in the interventricular septum and left ventricle after the 4-week treatment period. (a) The expression levels of CaMKII, p-CaMKII (Thr-286), PLB, RyR2, p-PLB (Thr-17), and FKBP12.6 in the interventricular septum of the four groups. (b) The expression of CaMKII, p-CaMKII (Thr-286), PLB, RyR2, p-PLB (Thr-17), and FKBP12.6 in the left ventricle of the four groups. (c) The expression level of CaMKII of the four groups in the interventricular septum and the left ventricle. (d) The expression level of Thr-286-phosphorylated CaMKII of the four groups in the interventricular septum and the left ventricle. (e) The expression level of PLB of the four groups in the interventricular septum and the left ventricle. (f) The expression level of Thr-17-phosphorylated PLB of the four groups in the interventricular septum and the left ventricle. (g) The expression level of RyR2 of the four groups in the interventricular septum and the left ventricle. (h) The expression of FKBP12.6 of the four groups in the interventricular septum and the left ventricle. (*P < 0.05 versus the MI group, ^▲^ *P* < 0.05 versus the sham group, and ^#^ *P* < 0.05 the amiodarone group versus the WXKL group).

Western blotting analysis was performed to examine the expression of CaMKII, p-CaMKII (Ser-286), PLB, p-PLB (Thr-17), RYR2, and FKBP12.6 in different areas of the myocardium among the four experimental groups (*n* = 5 per group).  Figure 3 shows the expression of these proteins in the interventricular septum and left ventricular areas (Figures 3(a) and 3(b)). As shown in Figure 3(c), the expression of CaMKII was increased in the MI group, the WXKL group, and the amiodarone group compared with the sham group (*P* < 0.05). Compared with the MI group, the expression of CaMKII was reduced in both the WXKL group and the amiodarone group (*P* < 0.05), while no significant differences were observed between the expression levels detected in the WXKL group and the amiodarone group (*P* > 0.05). No differences were detected in the expression levels of CaMKII between the interventricular septum and left ventricular areas (*P* > 0.05). As shown in Figure 3(d), phosphorylation of CaMKII at Thr-286 was increased in the MI group, the WXKL group, and the amiodarone group compared with the sham group (*P* < 0.05). However, when compared with that of the MI group, the expression level of Thr-286-phosphorylated CaMKII was significantly reduced in both the WXKL group and the amiodarone group (*P* < 0.05). Again, there were no statistically significant differences between the WXKL group and the amiodarone group in terms of their expression of Thr-286-phosphorylated CaMKII (*P* > 0.05). No statistically significant differences were observed in the expression of Thr286-phosphorylated CaMKII between the interventricular septum and the left ventricular areas (*P* > 0.05). The expression of PLB was significantly increased in the MI group and the amiodarone group compared with the sham group (*P* < 0.05), but the differences were not significant between the WXKL group and the sham group (*P* > 0.05) (Figure 3(e)). Compared with that seen in the amiodarone group, the PLB expression level in the WXKL group was significantly reduced (*P* < 0.05). In our analysis of the levels of phosphorylated PLB protein in the four experimental groups (Figure 3(f)), phosphorylation of PLB at Thr-17 was decreased in the MI group, the WXKL group, and the amiodarone group compared with the sham group (*P* < 0.05), but treatment with WXKL or amiodarone was found to significantly increase the level of Thr-17-phosphorylated PLB compared with that detected in the MI group (*P* < 0.05). As shown in Figure 3(f), the expression of RyR2 was significantly decreased in the MI group, the WXKL group, and the amiodarone group compared with the sham group (*P* < 0.05). Compared with that of the MI group, the RyR2 expression levels detected in rats treated with either WXKL or amiodarone were significantly increased (*P* < 0.05), but no significant differences in RyR2 levels were observed between the WXKL group and the amiodarone group (*P* > 0.05). When compared with that of the sham group, FKBP12.6 expression levels were significantly reduced in the MI group, the WXKL group, and the amiodarone group (*P* < 0.05). As was observed for RyR2 expression, treatment with either WXKL or amiodarone was found to significantly increase the expression of FKBP12.6, compared with levels detected in untreated MI group rats (*P* < 0.05), but no statistically significant differences were observed between the WXKL-treated group and the amiodarone-treated group (*P* > 0.05).

### 3.4. Effects of WXKL Treatment on Calcium Transients and SR Ca^2+^ Content *In Vitro*

**Figure 4:**
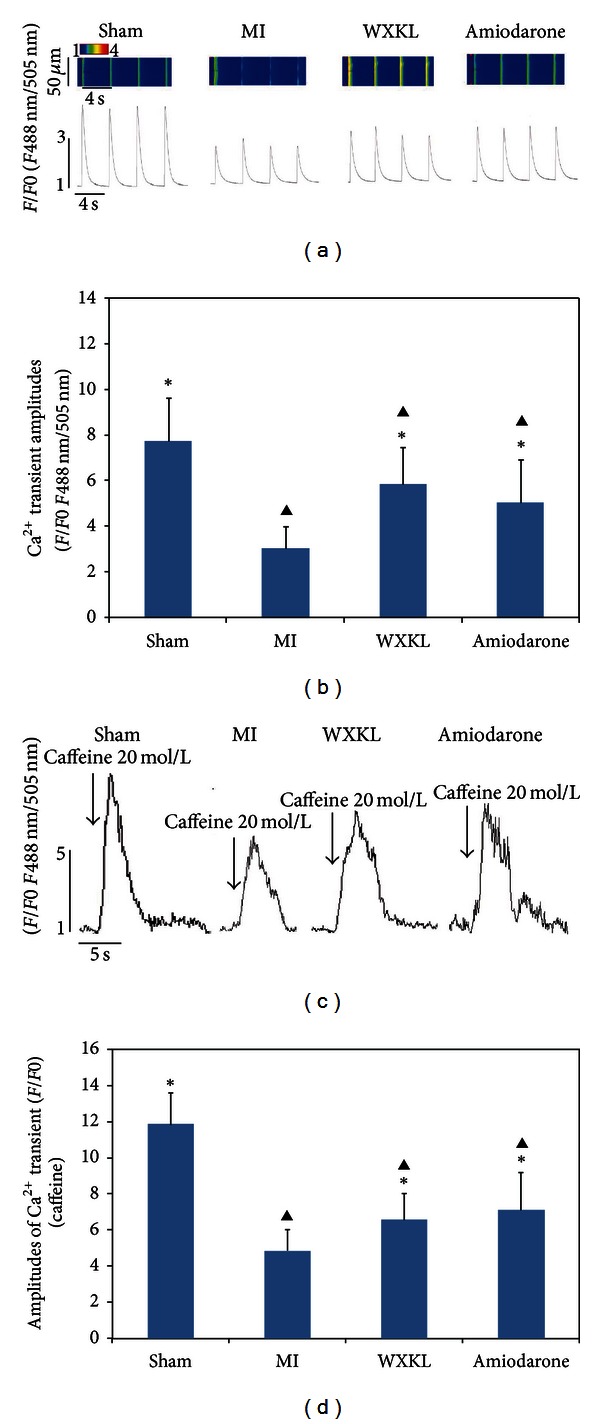
Effects of WXKL on the calcium transient and the Ca^2+^ content in the SR measured *in vitro *at 4 weeks after treatment. (a) The calcium transient amplitude was measured by stimulating the cultured adult cardiomyocytes at 0.5 Hz. (b) Compared with the sham group, the calcium transient amplitude was reduced in the MI group, the WXKL group, and the amiodarone group (*P* < 0.05,  *n* = 15 cells per group). Compared with that of the MI group, the calcium transient amplitude was significantly elevated in the WXKL group and the amiodarone group (*P* < 0.05). (c) The Ca^2+^ content in the SR was measured by assessing caffeine-induced calcium release in cultured adult cardiomyocytes. (d) The integrative volumes of the Ca^2+^ transients in the WXKL-treated group and the amiodarone group were increased significantly compared with those of the MI group (*P* < 0.05,  *n* = 12 cells per group). (*P < 0.05 versus the MI group, ^▲^ *P* < 0.05 versus the sham group).

To verify the pathological relevance of the changes in the expression of CaMKII and Ca^2+^-handling proteins observed following myocardial infarction, we measured the calcium transient amplitude by stimulating cultured adult cardiomyocytes at a frequency of 0.5 Hz. The Ca^2+^ level data are reported here as *F*/*F*0, where *F*0 is the resting or diastolic fluo-4 fluorescence. As shown in Figures 4(a) and 4(b), the calcium transient amplitude was decreased in the MI group, the WXKL group, and the amiodarone group versus the sham group (*P* < 0.05; *n* = 15 per group). In comparison with the MI group, the WXKL group and the amiodarone group both exhibited significantly elevated calcium transient amplitudes of WXKL (*P* < 0.05; *n* = 15 per group), but there were no significant differences between the amplitudes measured in the WXKL-treated cardiac myocytes and those of the amiodarone-treated cells (*P* > 0.05; *n* = 15 per group).

We measured the Ca^2+^ content in the SR by assessing caffeine-induced calcium release in cultured adult cardiomyocytes. When Ca^2+^ release from the SR was triggered by the application of 20 mM caffeine, transient Ca^2+^ elevation was significantly decreased in the MI group versus the sham group (*P* < 0.05; *n* = 12 per group). As shown in Figures 4(c) and 4(d), the integrative volume of the Ca^2+^ transient was increased significantly in both the WXKL-treated group and the amiodarone-treated group compared with the MI group (*P* < 0.05; *n* = 12 per group).

### 3.5. Effects of WXKL on the Incidences of Early EADs and Delayed DADs *In Vitro*

**Figure 5:**
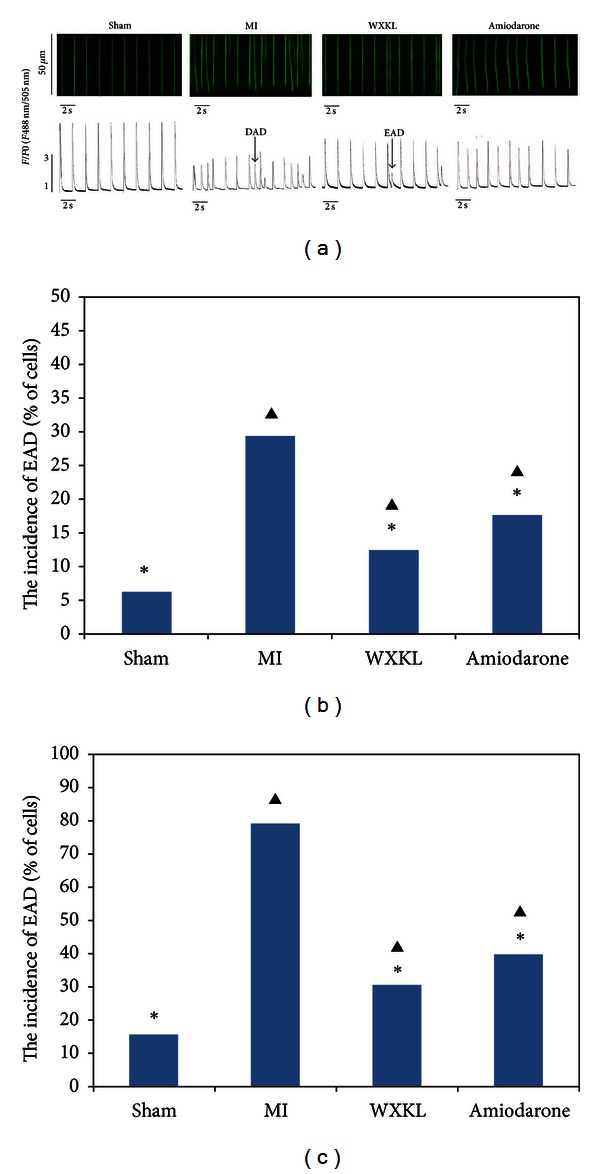
Effects of WXKL on the incidences of EADs and DADs *in vitro *at 4 weeks after treatment. (a) The incidences of EADs and DADs were recorded among the sham group, the MI group, the WXKL group, and the amiodarone group (*n* = 30 cells per group). (b) The incidence of EADs was significantly increased in the MI group compared with the sham group, the WXKL group, and the amiodarone group (29.4% versus 6.3%, 12.5% and 17.6%, respectively; *P* < 0.05 using Fisher's 2-sided exact test). (c) The incidence of DADs was significantly increased in the MI group compared with the sham group, the WXKL group, and the amiodarone group (79.1% versus 15.5%, 30.5% and 39.7%, respectively; *P* < 0.05 using Fisher's 2-sided exact test). (*P < 0.05 versus the MI group, ^▲^ *P* < 0.05 versus the sham group).

We isolated rat myocardial cells from all four experimental groups using enzymatic hydrolysis and measured the incidences of early EADsand delayed DADs in the cultured cells. The cardiomyocytes (*n* = 30 per group) were stimulated for 1 min at a frequency of 0.5 Hz and a pulse width of 2 s WXKL. As shown in Figure 5(a), nonstimulated Ca transients (EAD and DAD) were frequently observed in MI myocytes. EAD was characterised by a non-stimulated Ca increase before the turning point of the Ca transient decay, and this was accompanied by a minor cell contraction. In contrast, DAD occurred after the turning point of the Ca transient decay and was accompanied by a major contraction of the myocyte. Cardiac myocytes from the MI group were found to have the highest incidence of EADs (Figure 5(b); 29.4%, 6.3%, 12.5%, and 17.6% in the cells from the MI, sham, WXKL-treated, and amiodarone-treated groups, respectively; *P* < 0.05 using Fisher's 2-sided exact test, *n* = 30 per group) and of DADs (Figure 5(c); 79.1%, 15.5%, 30.5%, and 39.7% in the cells from the MI, sham, WXKL-treated, and amiodarone-treated groups, respectively; *P* < 0.05 using Fisher's 2-sided exact test, *n* = 30 per group) of WXKL.

### 3.6. Effects of WXKL on the Incidence of Cardiac Arrhythmias *In Vivo*

**Figure 6:**
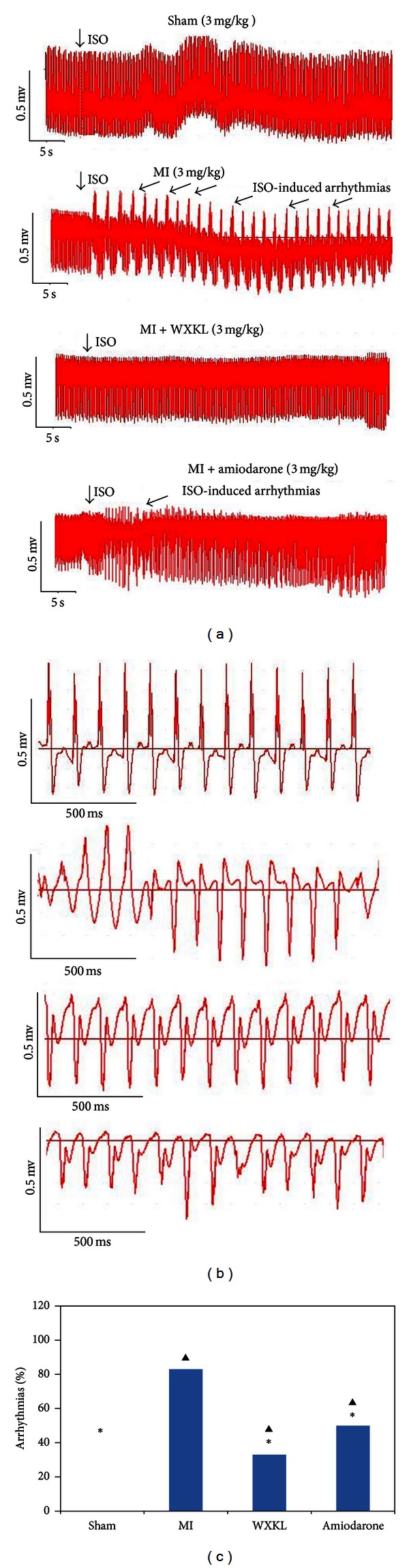
Effects of WXKL on the incidence of cardiac arrhythmias *in vivo*. (a) The ECG recordings from the four experimental groups following an intraperitoneal injection of ISO (3 mg/kg body weight). (b) Detailed tracings for the respective arrhythmic events. (c) WXKL treatment significantly reduced cardiac arrhythmias *in vivo* compared with the MI group and the amiodarone group (*P < 0.05 versus the MI group, ^▲^ *P* < 0.05 versus the sham group, and ^#^ *P* < 0.05 the amiodarone group versus the WXKL group, using Fisher's 2-sided exact test, 6 rats were analysed per group).

Our observation, made at the cellular level, of the antiarrhythmic effects of CaMKII inhibition prompted us to test whether WXKL treatment is sufficient to reduce the incidence of cardiac arrhythmias *in vivo*. The results of ECG recordings made following an intraperitoneal injection of ISO (3 mg/kg body weight) into rats from each of the four experimental groups are shown in Figure 6(a), while Figure 6(b) shows detailed tracings for representative arrhythmic events that are likely to correspond to previously described bidirectional tachycardias. The data summarised in Figure 6(c) shows that the WXKL group exhibited a significantly reduced incidence of cardiac arrhythmias *in vivo* when compared with the MI group and the amiodarone group. Two of 6 rats in the WXKL-treated group exhibited arrhythmias in the first 8 minutes after ISO application, while 5 of 6 rats in MI group and 3 of 6 rats in the amiodarone group exhibited arrhythmias during the same period. This difference was found to be statistically significant (*P* < 0.05 using Fisher's exact test).

### 3.7. Effects of WXKL on Ca Transient Amplitudes in ISO-Stimulated Cardiac Myocytes *In Vitro*

At high stimulation rates or in the presence of *β*-adrenergic stimulation, SR Ca load and [Ca]i increase substantially and may induce further arrhythmogenic triggers that could be a highly influential factor in the genesis of arrhythmias *in vivo*, and even under pathophysiological conditions such as in heart failure, catecholamine levels are known to be increased. Therefore, we decided to challenge the myocardial infarction cells using ISO (up to 10^−6 ^M) to load their SR Ca stores and further unmask their potential to exhibit diastolic proarrhythmogenic events.

**Figure 7:**
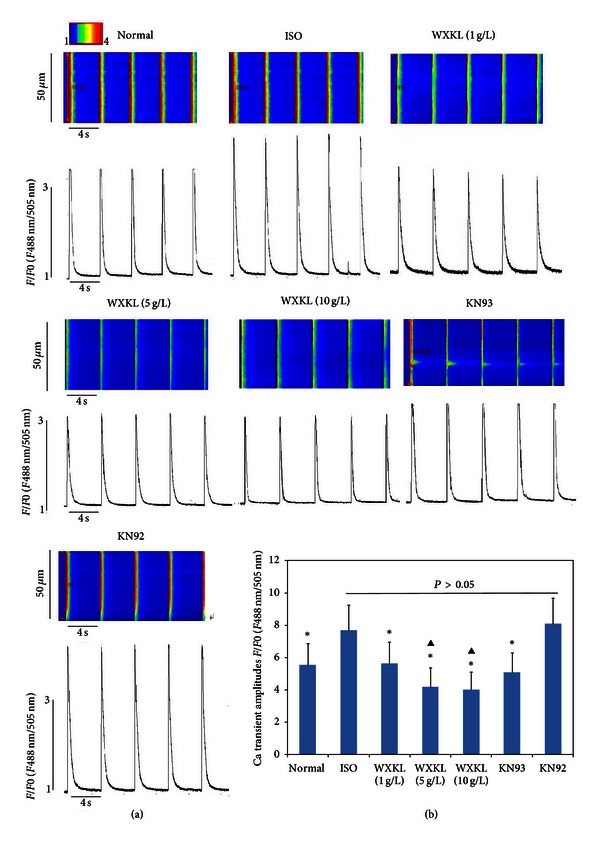
Effects of WXKL and KN93 on the Ca transient amplitudes in ISO-stimulated cardiac myocytes. (a) Detailed tracings for 7 treatment groups. (b) The Ca transient amplitudes in ISO-stimulated cardiac myocytes (10^−6 ^M ISO) were significantly increased compared with those in non-ISO-stimulated cells, but WXKL treatment (1 g/L, 5 g/L and 10 g/L) reduced the Ca transient amplitudes in a dose-dependent manner. Treatment with KN93 (1 mM) also significantly reduced the Ca transient amplitudes, while treatment with KN92 (1 mM) did not reduce them. (*P < 0.05 versus the ISO group,  ^▲^ *P* < 0.05 versus the normal group).

We first examined isolated normal myocytes using epifluorescence microscopy under basal and ISO-stimulated conditions (10^−6 ^M ISO). As shown in Figures 7(a) and 7(b), the Ca transient amplitudes in cardiac myocytes under ISO-stimulated conditions were significantly increased compared with those of nonstimulated cells, but treatment with WXKL at doses of 1 g/L, 5 g/L, and 10 g/L reduced the Ca transient amplitudes in a dose-dependent manner. Treatment of cardiac myocytes with KN93 (1 mM) also significantly reduced the Ca transient amplitudes to a degree similar to that achieved by WXKL treatment, while treatment with KN92 (1 mM) did not produce a significant reduction in amplitudes.

## 4. Discussion

We can draw the following conclusions from the present study. (1) WXKL treatment significantly improves cardiac function and inhibits myocardial remodelling. (2) In rats with myocardial infarction, WXKL treatment can significantly reduce the expression of CaMKII, p-CaMKII (Thr-286), and PLB, while significantly increasing the expression of RyR2_,_ p-PLB (Thr-17), and FKBP12.6. (3) WXKL can significantly increase both the Ca^2+^ content of the SR and the calcium transient amplitude in cultured cardiac myocytes from rats with myocardial infarction. (4) WXKL treatment can significantly decrease the incidences of EADs and DADs in myocardial infarction cardiomyocytes. (5) WXKL treatment can reduce the incidence of cardiac arrhythmias in rats with myocardial infarction. (6) WXKL can significantly suppress the Ca transient amplitudes in ISO-stimulated cardiac myocytes *in vitro*.

CaMKII is an appealing potential target for pharmacological inhibition. CaMKII activity is upregulated in hypertrophy and heart failure [22]. CaMKII overexpression in transgenic mice results in heart failure and arrhythmias [23, 24], whereas CaMKII inhibition protects the heart against the development of these conditions [25]. In the present study, we demonstrated that WXKL significantly decreased both the expression of CaMKII and its phosphorylation at Thr-286 in rats with myocardial infarction. When its functional effects were examined, WXKL was found to significantly improve cardiac function and inhibit myocardial remodelling. The observed decrease in both the expression of CaMKII and its phosphorylation at Thr-286 may be the primary mechanism by which WXKL inhibits heart failure and arrhythmia. 

Dissociation of FKBP12.6 from RyR channels causes uncoupled channel gating, which results in defective closure of these channels [26, 27]. In a study of the mechanism underlying the partial loss of FKBP12.6 from RyR channels, Marx et al. [15] demonstrated that hyperphosphorylation of RyR causes the dissociation of FKBP12.6 from the ion channel and that this causes an increased sensitivity to Ca^2+^-induced activation and defects. These findings suggest that failing hearts lack the normal FKBP12.6-mediated regulation of the RyR-family of ion channels and that this is the major cause of the serious abnormality in the regulation of intracellular Ca^2+^ and the consequent cardiac dysfunction. In line with our findings, Okuda et al. [28] demonstrated that hyperphosphorylation of the ryanodine receptor by PKA results in the channel exhibiting an abnormal Ca^2+^ leak and is associated with a decrease in the amount of ryanodine receptor-bound FKBP12.6. Treatment with WXKL or amiodarone significantly increased the expression of RyR2 and FKBP12.6 in rats with myocardial infarction, which may enhance the ability of FKBP12.6 to modulate the RyR2 ion channel. This may be the most important mechanism by which WXKL is able to inhibit arrhythmia. 

Until recently, there was a general agreement that CaMKII phosphorylates PLB at Thr-17 and that this leads to an improved frequency-dependent acceleration of relaxation [29]. It was reported that the mRNA and protein expression levels of PLB were significantly upregulated by 55.5% (*P* < 0.05) and 84.8% (*P* < 0.01), respectively, in rats of the chronic heart failure (CHF) group following ligation of the coronary artery for 6 weeks [30]. In the present study, we demonstrated that WXKL significantly decreased the expression of PLB, but increased the level of Thr-17-phosphorylated PLB in the final stage of heart failure (Figure 3). By increasing the level of PLB, that is, phosphorylated at Thr-17, and decreasing the expression level of total PLB, WXKL may improve the frequency-dependent acceleration of relaxation in cardiac myocytes, thereby improving cardiac function and preventing arrhythmia. 

In heart failure (HF), where CaMKII expression and activation are increased, RyR phosphorylation and the diastolic SR Ca leak are also increased [12], and this diastolic SR Ca leak can initiate DADs in which the depolarising current consists of an inward Na/Ca exchange. Studies in genetically modified animal models provide proofs of concept that this type of CaMKII-modified RyR behaviour can be a major arrhythmogenic factor that promotes HF and atrial fibrillation [31, 32]. In the present study, treatment with either WXKL or amiodarone clearly decreased the incidence of DADs and increased both the Ca^2+^ content in the SR and the calcium transient amplitude. It is possible, therefore, that the decreased incidence of DADs that followed treatment with WXKL or amiodarone was the result of a decrease in the diastolic SR Ca leak. 

The results of this study demonstrate that myocardial infarction-induced overexpression of CaMKII increases the incidence of cellular proarrhythmogenic events. It has been reported elsewhere that CaMKII activity is associated with the generation of these systolic proarrhythmogenic events [24, 33]. CaMKII activity can contribute to L-type Ca current facilitation [23, 25] and may therefore favour EAD generation [24, 33]. However, the results of the present study demonstrate that, as shown in Figure 6, treatment with WXKL or amiodarone can reduce the incidence of EADs. By inhibiting the expression of CaMKII and p-CAMKII (Thr-286), WXKL and amiodarone may affect the function of LTCC and reduce systolic calcium influx into the cell, thereby inhibiting arrhythmia.

It should be noted that ISO may also activate CaMKII directly through exchanging protein directly activated by cAMP-dependent pathways and indirectly through increasing [Ca]i [34], thus resulting in more dramatic CaMKII-dependent cellular arrhythmias *in vitro*. The results of this study demonstrate that the Ca transient amplitudes in ISO-stimulated cardiac myocytes were significantly increased compared to those of non-ISO-stimulated cells. In our experiments examining the effect of WXKL on Ca transients, the Ca transient amplitudes decreased with increasing WXKL dose. Treatment with KN93, a specific inhibitor of CaMKII, was also found to significantly reduce the Ca transient amplitudes in cardiac myocytes, while treatment with KN92, which has the same structure as KN93 but no CaMKII-inhibiting activity, had no effect. It may be that WXKL by inhibiting CaMKII activity reduces [Ca]I and thereby prevents the occurrence of arrhythmia. 

## 5. Conclusions

 In summary, the present study shows that WXKL and amiodarone inhibit heart failure and cardiac arrhythmias via a mechanism that may involve the regulation of the CaMKII signal transduction pathway. WXKL treatment significantly reduced the expression of CaMKII, p-CaMKII (Thr-286), and PLB but significantly increased the expression of RYR2, p-PLB (Thr-17), and FKBP12.6 in rats with myocardial infarction to improve cardiac function and inhibit myocardial remodelling. While it was found to suppress the Ca transient amplitude in ISO-stimulated cardiac myocytes, WXKL increased both the SR Ca^2+^ content and the calcium transient amplitude in isolated cardiac myocytes from rats with myocardial infarction, while also significantly decreasing the incidences of EADs and DADs in these cells. Furthermore, WXKL significantly reduced the incidence of cardiac arrhythmias in our *in vivo* rat myocardial infarction model.
